# Autaptic Connections Shift Network Excitability and Bursting

**DOI:** 10.1038/srep44006

**Published:** 2017-03-07

**Authors:** Laura Wiles, Shi Gu, Fabio Pasqualetti, Brandon Parvesse, David Gabrieli, Danielle S. Bassett, David F. Meaney

**Affiliations:** 1 Department of Bioengineering, School of Engineering and Applied Sciences, University of Pennsylvania, Philadelphia, PA 19104, USA; 2 Applied Mathematics and Computational Science Graduate Program, School of Arts and Sciences, University of Pennsylvania, Philadelphia, PA 19104, USA; 3 Department of Mechanical Engineering, University of California Riverside, CA, USA; 4 Department of Electrical and Systems Engineering, School of Engineering and Applied Sciences, University of Pennsylvania, Philadelphia, PA 19104, USA; 5 Department of Neurosurgery, Perelman School of Medicine, University of Pennsylvania, Philadelphia, PA 19104, USA

## Abstract

We examine the role of structural autapses, when a neuron synapses onto itself, in driving network-wide bursting behavior. Using a simple spiking model of neuronal activity, we study how autaptic connections affect activity patterns, and evaluate if controllability significantly affects changes in bursting from autaptic connections. Adding more autaptic connections to excitatory neurons increased the number of spiking events and the number of network-wide bursts. We observed excitatory synapses contributed more to bursting behavior than inhibitory synapses. We evaluated if neurons with high average controllability, predicted to push the network into easily achievable states, affected bursting behavior differently than neurons with high modal controllability, thought to influence the network into difficult to reach states. Results show autaptic connections to excitatory neurons with high average controllability led to higher burst frequencies than adding the same number of self-looping connections to neurons with high modal controllability. The number of autapses required to induce bursting was lowered by adding autapses to high degree excitatory neurons. These results suggest a role of autaptic connections in controlling network-wide bursts in diverse cortical and subcortical regions of mammalian brain. Moreover, they open up new avenues for the study of dynamic neurophysiological correlates of structural controllability.

Network architecture forms a critical driver for complex function in a broad class of systems across biomedical science^1^,^2^,^3^. For the brain, transcribing the architecture of white matter tracts crisscrossing the human cortex^4^,^5^ have offered inherently new ways to explain the relationship between brain and behavior^6^,^7^, and its alteration in neurological disorders and psychiatric disease^8^,^9^,^10^,^11^. At the microcircuit scale, describing the anatomical connections among neurons in a network has shown that networks do not follow a random organizational pattern within the brain, but instead follow a clustered, distance-dependent connection pattern that provides for self-sustained excitability within a cluster of neurons^12^,^13^,^14^, the coordinated synchronization of activity across clusters^15^,^16^, and reveals important synaptic scaling features to organize these neuronal circuits across the phylogenic scale^17^. In each of these contexts, the organization of connectivity patterns plays a key role in constraining system dynamics and organism function^18^.

In neural networks, self-looping structures are known as “autapses” may offer energetically effective means for controlling network dynamics towards specific states^19^. Autapses appear as a synaptic connection from a neuron onto itself. Since their discovery over four decades ago, autapses are now documented in pyramidal neurons within the developing rat neocortex^20^ and the cat visual cortex^21^, appear more commonly on inhibitory neurons^22^,^23^,^24^, and appear abundantly in fast-spiking interneurons, but not in low-threshold spiking interneurons^23^. In other cases, autaptic connections can represent only a small number of the thousands of excitatory and inhibitory synaptic connections received by a neuron^25^, yet their self-stimulating nature can provide a very economical method to affect neuronal activity dynamics. To this end, several studies show that the delays in autaptic inputs affect the bursting behavior and information transfer of individual neurons, offering insights into regulating the activity of these neurons^26^,^27^,^28^,^29^. However, relatively little is known on the consequences of self-loop connections at the network scale, and how these connections affect the overall dynamics of the neural network. As such, principles explaining the functional network role of autapses in neural circuits remain a mystery.

In this communication, we study self-looping in cortical and hippocampal neuronal networks and examine the impact of these loops on activity dynamics, from network-wide bursting to coordinated firing of neuronal subpopulations. We test the specific situation in which self-loops are placed either randomly throughout the network or at driver nodes predicted to facilitate different control strategies. We show that autaptic connections enhance the network’s excitability, increasing bursting frequency and regularity. For the networks studied, effects of autaptic connections are strongest when these connections are placed on excitatory neurons; when the number of autaptic connections is held constant, bursting frequency is higher when more autapses are placed on fewer neurons rather than when fewer autapses are placed on more neurons. Finally, we observed the greatest increase in network-wide bursting when autapses were located at points in the network that are theoretically predicted to be effective controllers.

## Methods

To study the relationship between structural connectivity and neuronal network dynamics, we constructed computational neural networks and simulated their activity using Izhikevich integrate and fire model neurons^30^,^31^. Preliminary simulations showed that network activity dynamics did not change for networks containing more than 800 neurons. We therefore used a network size of 1000 neurons for all simulations. All simulations were completed using in-house software developed in the MATLAB programming language (MathWorks, Inc.).

### Neural Network Simulations: Neurons and Their Coupling

In each simulation, we placed 800 excitatory and 200 inhibitory neurons on the surface of a unit sphere using MATLAB’s twister random number generator. We used a uniform distribution to place these neurons at different azimuthal and polar angles and to avoid clustering of the neurons at either pole. The number of outputs for each neuron was generated from a normal distribution with a mean of 93.75 outputs per neuron and a variance of 9.375, resulting in a mean of 75 excitatory inputs and 18.75 inhibitory inputs per neuron. We chose these values to reflect anatomical estimates from empirical data that suggest that – in cortex – approximately 20% of neuron inputs are inhibitory^32^. We placed these output and input connections within the network in a distance-dependent manner, consistent with prior empirical studies^33^,^34^. We defined the strength of each neuron as the sum of inputs onto and outputs from a neuron. For example, an inhibitory neuron with 100 excitatory inputs (each strength 3) and 200 inhibitory outputs (each strength −5) would show a total neuron strength of −700.

We connected outputs from each neuron to other neurons using a distance-dependent drop-off probability function *P*_*ij*_ = *1*/*d^2^*, where *d* is the arc length between node *i* and node *j* along the surface of the sphere. Collectively, these connections between all possible pairs of nodes formed the connectivity matrix, ***A***. The weight of an edge, codified in the element *A*_*ij*_, represents an aggregate synaptic strength drawn from a normal distribution with a specified mean strength and a standard deviation of 0.1, consistent with prior work^35^,^36^,^37^,^38^. We used a standard deviation of 0.1 to maximize variance of connection weights while minimizing overlap among synaptic strengths from simulations with different mean strengths. For example, if we have two different networks with mean excitatory strengths of 2 and 3, with a standard deviation of 0.1, nearly all of the individual strengths will fall in the ranges of 1.7–2.3 and 2.7–3.3, respectively, which allows for a distribution of synaptic weights while maintaining the difference between these two networks. The diagonal elements of the weighted adjacency matrix ***A*** are equal to zero, representing the fact that there are no self-connections (or autapses) present.

### Neural Network Simulations: Model of Dynamics

We model neuronal activity with systems of ordinary differential equations, following the work of Izhikevich in 2003. First, we define the neuron’s membrane potential, membrane recovery variable, and after-spike reset values as follows:


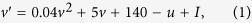



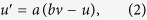



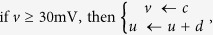


where the dimensionless variable *v* represents the neuron’s membrane potential and *u* represents the neuron’s membrane recovery variable. Each neuron is assigned the parameters *a, b, c*, and *d*, which govern the intrinsic behaviors and dynamics of the neurons^30^. For our simulations, we assigned values for *a, b, c*, and *d* such that the behavior of excitatory neurons would be characterized by regular-spiking, consistent with the majority of neurons in the cortex, but still exhibit enough heterogeneity that any two neurons would never display identical dynamics^30^. For inhibitory neurons, values for *a, b, c*, and *d* were assigned such that both fast-spiking and low-threshold spiking interneurons existed in the simulated system^30^.

Following^31^, we applied a random thalamic input to the network of 1 Hz, consistent with the mean firing rates of cortical neurons observed *in vivo*^39^,^40^. We included the exponential decay of synaptic currents. The rate of membrane potential change was capped (225 mV/ms) to avoid unrealistic membrane potentials (>50 mV) during a spiking event. When a neuron fired an action potential, the current was injected into output neurons in the next time step (0.2 ms later).

### Normative Dynamics

We studied neuronal dynamics in networks characterized by different excitatory and inhibitory strengths to identify excitation and inhibition levels that produced similar spiking activity. Intuitively, at different excitation and inhibition levels, a neuron might require fewer or more synchronous inputs to fire an action potential. We examined 10 mean excitatory strengths, from 1 to 10 in unit increments. With a mean excitatory connection strength of 1, a neuron would need to receive approximately 20 synchronous inputs to fire an action potential; with an excitatory connection strength of 10, a neuron would only need to receive two synchronous inputs to fire an action potential^31^. To complement these 10 excitation levels, we also examined 10 mean inhibitory strengths, from −10 to −1 in increments of unity. To achieve numerical stability and obtain robust results, we performed ten 120 s stimulations with 5 steps/ms for each possible combination of mean excitatory strength and mean inhibitory strength. We then analyzed the resultant spiking behavior to measure firing rate and to isolate bursts.

### Addition of Autapses

**Figure 1:**
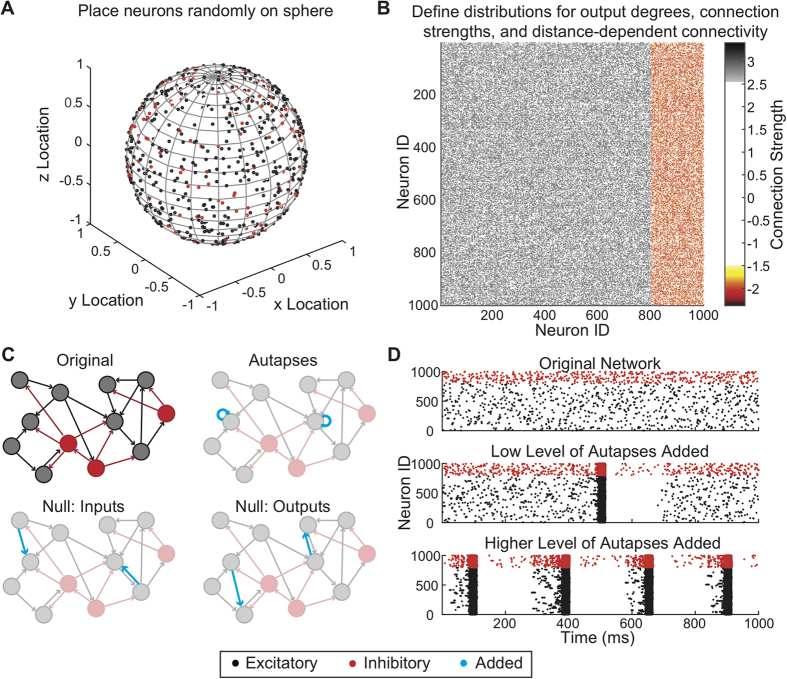
Schematic of Empirical Methods. (**A**) Excitatory and inhibitory neurons were placed uniformly at random on the surface of a sphere. (**B**) Coupling between neurons was constructed using well-defined distributions for node strength, and by implementing a set probability of connection fall-off with distance. The resulting coupling matrix is a weighted, directed adjacency matrix. (**C**) Autapses were added to either inhibitory or excitatory neurons to determine their effect on network dynamics, in comparison to appropriate statistical null models where input or output connections were added in place of autapses. (**D**) We measured the effects of autapses on the dynamics of the network activity by quantifying firing rate and network-wide bursts.

We added autapses, defined as self-loops in the network (mathematically: non-zero elements on the diagonal of the connectivity matrix ***A***), to either excitatory or inhibitory neurons using two characteristics of that node’s connections: strength and controllability (Fig. 1C,D). Strength is defined as the sum of the inputs onto and outputs from that neuron. Controllability can be separated into notions of average control and modal control, which are defined in detail in the next section. Here we simply describe these notions intuitively. Average control describes the theoretically predicted preference for the node to push the system into local easily-reachable states, and modal control describes the theoretically predicted preference for the node to push the system into distant difficult-to-reach states. Strength, average control, and modal control provide complementary estimates of the influence a node has on network dynamics.

When adding autapses, we implemented seven targeting strategies, adding autapses to neurons with the (1) highest strength, (2) lowest strength, (3) highest average controllability, (4) lowest average controllability, (5) highest modal controllability, and (6) lowest modal controllability as well as (7) neurons chosen uniformly at random. To construct appropriate null models for our subsequent analyses, we consider that by adding autapses to a neuron, we increased both the number of output connections from and the number of input connections to the selected neuron. We therefore implemented two null models. First, we constructed a null model that accounts for the increase in outputs on autaptic neurons by adding outputs from the would-be autaptic neurons to other neurons in the network in a distance-dependent manner. Second, we constructed a null model that accounts for the increase in inputs on autaptic neurons by adding inputs from other neurons to the would-be autaptic neuron.

In both autaptic network and null models, we added connections (self-loops or non-self-loops, respectively) to between 10% and 100% (in increments of 10%) of either excitatory or inhibitory neurons. Given the relatively rare frequency of autaptic connections *in vivo*, we added small amounts of autaptic or non-autaptic connections (1%, 2%, 3%, 4%, 5%) to the selected neurons. Autaptic connections were added as self-looping connections in proportion to the outputs from a given neuron; e.g., a neuron with 100 separate outputs to other neurons received 3 additional self-looping (autaptic) connections to produce 3% new autaptic connections. Non-autaptic connections followed a similar mapping. To maintain consistency with previous simulations, current was injected from these autaptic connections at the next timestep. To obtain robust results, we completed ten sets of simulations for each combination of excitatory and inhibitory strengths. We performed each of the ten simulations at a given excitation and inhibition level on different original connectivity matrix with no autapses, which we then modified by adding autaptic or non-autaptic connections using the targeting strategies described above. From each modified network, we analyzed spiking activity to better understand the effects of targeted connectivity changes on bursting behavior.

### Targeting Strategies

We employ the targeted addition of autapses to neurons to study potential mechanisms by which a network can control its global dynamics. The simplest notion of a node that has a high level of influence on dynamics is the notion of a hub^41^. A hub is a node that has either many connections emanating from it (high degree), or on average very strong connections emanating from it (high strength) or both. Here because we are studying inherently weighted graphs, we study a neuron’s strength, defined as the sum of the inputs onto and outputs from that neuron. In prior studies, this metric of influence has been shown to be an indirect proxy for controllability^42^,^43^ and to correlate with statistical measurements of system dynamics^44^,^45^,^46^.

Arguably a more direct measure of influence is one that would consider not just which connections a neuron had, but also how the neuron used them. Philosophically, the notion of *influence* is essentially a *dynamical* notion, implying change in a system’s state. Thus, for a more direct measure of influence, we turned to applications of dynamical systems theory to the problem of network control. In network control theory, one wishes to understand how to drive a networked system from a specified initial state to a specified target state in finite time and with limited energy. The rather nascent field has developed a theoretical framework, analytical results, and statistical tools that can be used to identify control points, which are theoretically predicted to be critical for driving the network’s observed dynamics^19^,^47^.

Traditionally utilized to study technological, robotic, and mechanical systems, network control theory offers a particularly appealing conceptual and mathematical framework in which to study neural systems^42^,^48^,^49^. In this context, control points in the network are neurons that are predicted to be critical for driving large-scale neural dynamics. To identify these control neurons, we implement a linearized generalization of nonlinear models of cortical activity^50^,^51^. Specifically, we used a noise-free linear discrete-time and time-invariant model of network dynamics^42^,


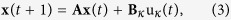


where **x** describes the state of neurons over time, and **A** is a signed, weighted, directed adjacency matrix whose elements, *A*_*ij*_, specify the strength of the connection from node *i* to node *j* (after a normalization to ensure Schur stability). The matrix **B**_κ_ is an input matrix that identifies the control neurons, *κ* = {*k*_*1*_, …, *k*_*m*_}, such that B_κ_ = [*e*_*k1*_ … *e*_*km*_], where *e*_*i*_ notes the *i*-th canonical vector of dimension N, and N is the number of neurons in the network. The signal u_κ_ is the control input to the control neurons.

Using this model, we can define two distinct controllability strategies: the average controllability and the modal controllability, which – as mentioned earlier – describe the ability to push a system into local easily-reachable states or into distant difficult-to-reach states, respectively. To define the notion of average controllability, we first write down the controllability Gramian, **W**_*κ*_, as


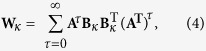


where T indicates a matrix transpose and τ is a constant ranging from 0 to infinity. Then, average controllability is defined as the trace of the inverse of the controllability Gramian Trace (**W**_*κ*_^−1^), but for computational purposes can also be approximated via Trace **(W**_*κ*_^−1^) (see ref. 42). Thus, to identify nodes with the highest average controllability, we select nodes that maximize Trace **(W**_*κ*_). Since the trace is a linear mapping and is invariant under cyclic permutations, we note that


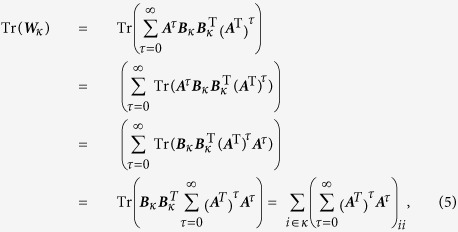


where 
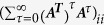
 represents the *i-*th diagonal entry of the matrix 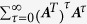. To maximize the trace, we chose the set of nodes, κ, containing the largest diagonal entries of 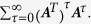 If **A** is stable, then ***X*** = 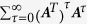 is the solution to the discrete-time Lyapunv equation, ***AXA***^T^ − ***X*** + ***Q*** = 0, where ***Q =  ***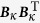. We then assign a ranked value of average controllability between 1 and N to each neuron, with 1 representing the neuron with the lowest average controllability and N representing the neuron with the highest average controllability.

To complement the notion of average control, we also define modal controllability, which is highest in nodes that can steer the system toward difficult-to-reach states. Modal controllability is calculated from the eigenvector matrix ***V*** = [*v*_*ij*_] of the connectivity matrix **A**, where *v*_*ij*_ measures the controllability of mode λ*j(**A***) from control node *i*. We can then define a scaled measure of the controllability of all N modes, *λ*_*1*_*(**A**), …, λ*_*N*_*(**A**)*, from neuron *i* as:


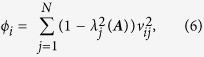


We assign each neuron a ranked value between 1 and N based on their ϕ value, with 1 being the neuron with the lowest modal controllability (lowest ϕ) and N being the neuron with the highest modal controllability (highest ϕ).

### Neuronal Activity and Network-Wide Bursts

Now that we have defined strategies to target the addition of autapses to neurons, we wish to understand their role in controlling global network dynamics. We therefore define several summary statistics of neural dynamics including firing rate and network-wide bursts, which are coordinated firing events across large numbers of neurons within a brief time period. Note that other complementary definitions of what consists a network-wide burst can be found in the literature, and we briefly review them in the Supplement.

Quantitatively, we define network-wide bursts as periods of activity in which the number of neurons firing at the same time met or exceeded a threshold level of 40% of neurons in a millisecond. We implemented a 5 ms tolerance in the burst detection algorithm, combining two groups of neurons into a single burst if they fired within 5 ms of each other. This burst detection algorithm was robust to changes in the threshold level of neurons that must be active for a period of activity to be considered a burst (Supp. Fig. 1).

After defining bursts, we calculated the mean and standard deviation of three summary statistics for each simulation: the burst frequency, the interburst-interval, and the burst duration. We defined the burst frequency to be the mean number of bursts per second across the simulation. We calculated the mean interburst-interval (IBI) from the temporal midpoints of the bursts in each simulation. Finally, we defined the mean burst duration as the fraction of simulation time spent in the network-wide bursting state. To summarize these results across simulations, we calculated either an unweighted mean or standard deviation (for burst frequency) or a weighted mean or standard deviation (for burst duration and average IBI). We used a weighted mean for the second two statistics because each simulation was built on a different connectivity matrix, and therefore could display different bursting parameters. For the IBI and burst duration, we computed the weighted average, *X*_*weighted*_, as follows:


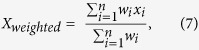


where *x*_*i*_ is the data value (the average IBI or burst duration from the individual simulation), the weight *w*_*i*_ = 1/*σ*_*i*_^2, *σ*_*i*_ is the standard deviation of *x*_*i*_ , and *n* is the number of data values (number of independent simulations). We also calculated the weighted standard deviation


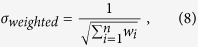


for the IBI and burst duration across simulations. Together, these parameters described the bursting behavior of the networks with and without autapses.

### Statistical Analysis

We used JMP Pro 11 (SAS Institute Inc.) for all statistical analyses. To identify autaptic conditions where the addition of autapses induced significant changes in burst frequency, we performed a mixed-model ANOVA using the Full Factorial Repeated Measures ANOVA Add-In (https://community.jmp.com/docs/DOC-6993, Julian Harris, SAS employee). Each original, non-autaptic connectivity matrix was treated as a “subject.” The between-subjects factor was the type of connection added (i.e., autapses, non-autaptic inputs, or non-autaptic outputs) while the within-subjects factor was the percent connections added, either autaptic or non-autaptic connections. When the interaction effect between the type of connection added and the percent connections added was significant, we performed post-hoc analyses using Tukey’s HSD test. These post-hoc results were used to identify targeting strategies and simulation parameters in which adding autapses significantly changed burst frequency from that of the original non-autaptic network and from the appropriate null models. Separate statistical analyses were completed for each of the seven targeting strategies and for each possible pair of excitatory and inhibitory strength values.

To identify differences in burst frequency induced by adding connections according to the seven targeting strategies, we again performed a mixed-model ANOVA using the Full Factorial Repeated Measures ANOVA Add-In (https://community.jmp.com/docs/DOC-6993, Julian Harris, SAS employee) where each original connectivity matrix was a “subject.” Here, the within-subjects factor was again the percent connections added. The between-subjects factor was the targeting strategy used to select neurons to which to add autaptic or non-autaptic connections. When there were significant effects of the interaction between the type of connection added and the targeting strategy, we performed post-hoc analyses using Tukey’s HSD test. These post-hoc results were used to identify the simulation parameters between which the burst frequency significantly differed. Additionally, when there were significant effects of the targeting strategy, we performed post-hoc analyses to identify which targeting strategies were significantly different from one another. In the main text, we report results comparing three targeting strategies: neurons chosen by highest average controllability, highest modal controllability, and uniformly at random. In the supplement, we show results for all seven targeting strategies (see Supp. Fig. 2). Separate statistical analyses were performed for each type of connection added within each excitatory/inhibitory strength combination.

## Results

### Network Construction

We modeled networks of cortical neurons by placing excitatory and inhibitory neurons on the surface of a sphere (Fig. 1A) and connecting them – with no autapses – in a distance-dependent manner (Fig. 1B). We next modified these networks by adding autapses (Fig. 1C), or self-loops, to groups of excitatory or inhibitory neurons chosen using certain characteristics of their local network neighborhood. We also constructed null model networks by adding input or output connections on the selected group of neurons to account for the increase in the number of connections in a network in which autapses were added (Fig. 1C). These null models were used to test whether the observed dynamical changes were simply due to the increase in connections in the autaptic networks or were more interestingly due to the autaptic nature of the connections specifically. We study the effect of autapses on several summary statistics of bursting behavior (Fig. 1D). See Materials and Methods for additional details.

### Network Dynamics

**Figure 2:**
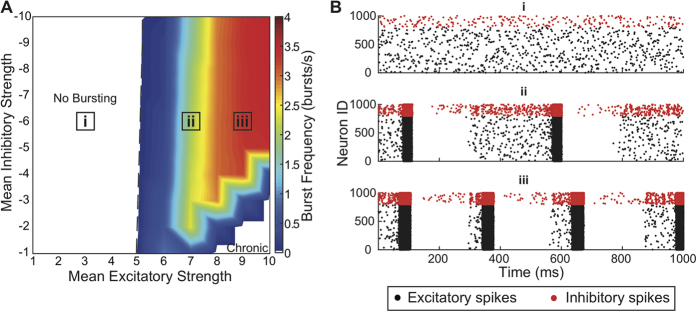
Dynamics of neuronal network simulations without autapses. (**A**) Burst frequencies at different excitatory and inhibitory strengths, showing regimes of no bursting [i], intermittent bursting [ii], and chronic bursting [iii]. (**B**) Example raster plots displaying activity at three different excitation levels.

We observed no change in dynamics if we simulated from 120 seconds to 3600 seconds of neural activity in the network. Similarly, we observed no change in the dynamics across networks of different size (1,000–10,000 neurons; see Supplemental Fig. 2). Therefore, we simulated 120 s of activity for networks with different levels of mean excitatory and inhibitory strength (Fig. 2). Based on the amount of excitation and inhibition present in the network, we identified three distinct regimes of neural dynamics. At low excitation levels, independent of inhibition level simulated, network-wide bursting never occurred (Fig. 2B,i). Activity in this regime was asynchronous and dominated by noise. As the excitation level increased, activity became less dependent on noise and network-wide bursts occurred more frequently and more regularly (Fig. 2B,ii). At high excitation levels, the network entered a chronic bursting regime (Fig. 2B,iii). The decrease in burst frequency at very high excitatory and very low inhibitory strengths is due to this transition to chronic bursting; burst frequency is decreasing as burst duration is increasing (see Supp. Fig. 3).

### Location of Control Points in the Network Depends on the Levels of Excitation and Inhibition

**Figure 3:**
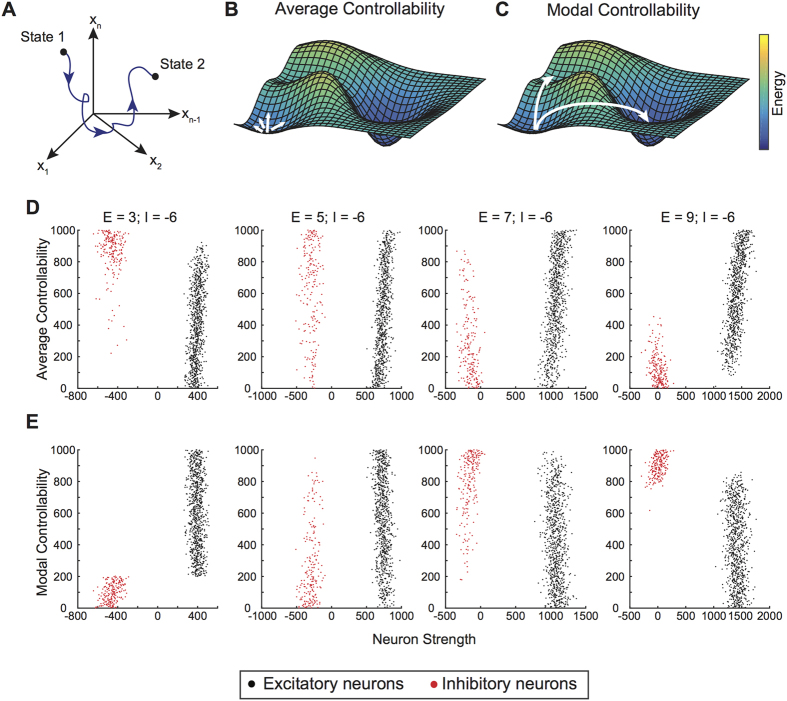
Notions of Network Control and Their Relationships to Topology. (**A**) Schematic illustrating the idea that a controllable network can be driven from an initial state to a final state in some multidimensional landscape within a finite time period. (**B**) Illustration of a 3-dimensional energy landscape on which nodes with high average controllability drive the system from a baseline state to many easily reachable states (arrows). One example of an easy to reach state is when a network is bursting at low, irregular frequency and transitions to a new, more regular bursting state. Alternatively, an easy to reach state for a nonbursting network is to maintain this nonbursting state. (**C**) Illustration of the same 3-dimensional energy landscape on which nodes with high modal controllability drive the system to difficult-to-reach states, from one energy minimum to another over a large energy barrier (arrows). An example of a difficult to reach state is when a bursting network transitions to a non-bursting network. (**D**) As excitatory synaptic strength in the network is increased while inhibitory strength is held constant, the nodes with the highest average controllability shift from the inhibitory to the excitatory network. At low excitatory levels, the network is not bursting and an easy to reach state is to maintain this nonbursting activity; hence the highest average controllability resides with the inhibitory network. (**E**) Over the same span of network configurations, highest levels of modal controllability appear in the excitatory network at low synaptic strength and shifts to the inhibitory network at high excitatory synaptic strengths. At high synaptic strengths, the network is bursting regularly and controllability of inhibitory neurons is key to bring the network to a difficult to achieve, nonbursting state.

Controllability describes the potential to drive a dynamical system from an initial state to a desired final state given that inputs are applied to one or more nodes in the network (Fig. 3A). The importance of individual nodes in driving the system to certain states can be quantified using distinct control strategies. Two commonly studied control strategies are average control and modal control. Nodes with high average controllability are theoretically predicted (by a simplified model of linear system dynamics) to drive the system to many energetically easy-to-reach states (Fig. 3B). Nodes with high modal controllability are theoretically predicted (again, by a simplified model of linear system dynamics) to drive the system to many difficult-to-reach states (Fig. 3C).

In the simulated networks, we found that average and modal controllability were directly related to node strength in excitatory neurons (Fig. 3D,E). In the excitatory population, we observe a positive correlation between average controllability and the total neuron strength, and a negative correlation between modal controllability and the total neuron strength. Moreover, we found these associations between controllability and neuron strength were driven primarily by the output strength of each neuron in the network. These relationships are consistent with those observed in undirected networks representing large-scale white matter connectivity in the human brain^42^,^48^.

Interestingly, the relationship between controllability and strength in the inhibitory neurons was less clear, as we observed a non-trivial dependence between the controllability statistics and the balance between excitation and inhibition in the network. Specifically, when the mean inhibitory strength was larger than the mean excitatory strength, excitatory neurons displayed lower average and higher modal controllability values, while inhibitory neurons displayed higher average and lower modal controllability values. In contrast, when the excitatory strength was larger than the inhibitory strength, excitatory neurons tended to display higher average and lower modal controllability values than inhibitory neurons. As the difference between the magnitudes of excitatory and inhibitory strength increased, the separation between controllability values of the excitatory and inhibitory neurons became more prevalent.

### Adding Autapses to Excitatory Neurons Increases Burst Frequency

**Figure 4:**
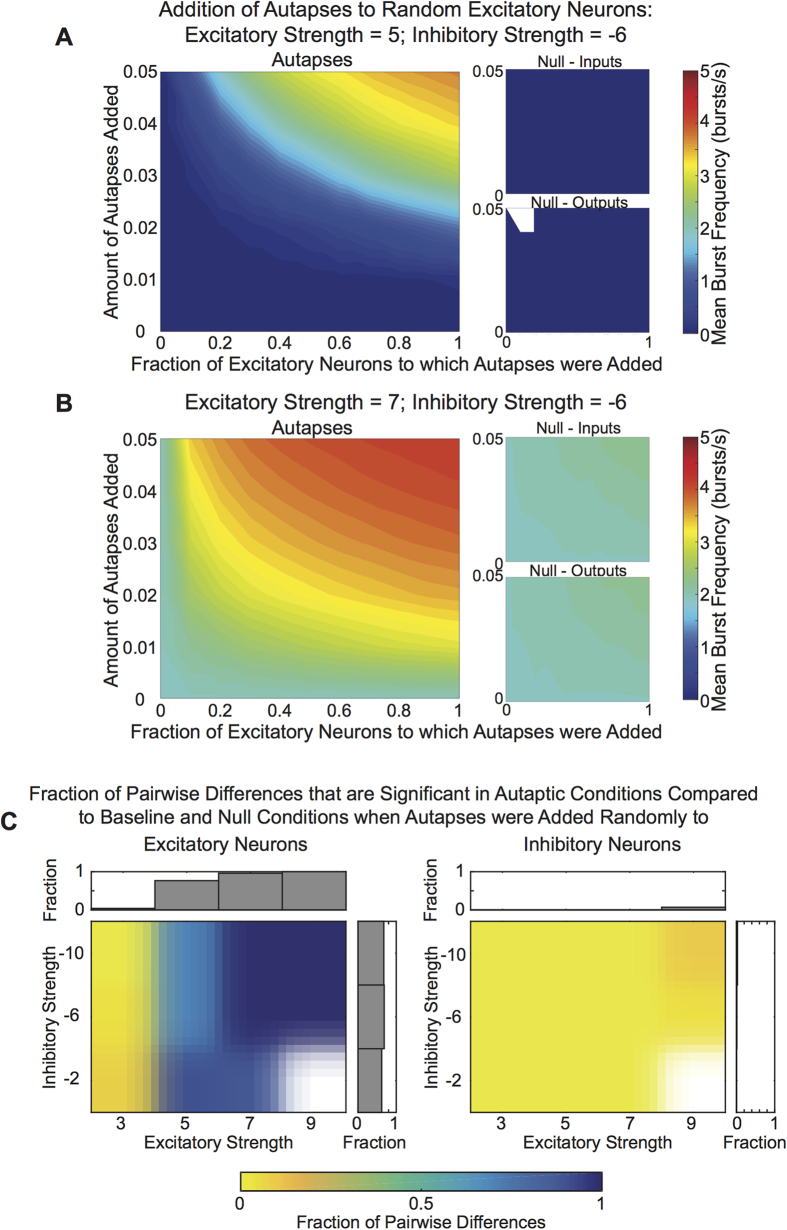
Role of Autapses on Excitatory Neurons in Network-Wide Bursts. (**A**) Burst frequency for an example simulation in the autaptic model (left) in which autaptic connections were added to excitatory neurons uniformly at random, or in two null models (right) in which non-autaptic connections were added to maintain either the number of input connections (an input null model) or to maintain the number of output connections (an output null model). This network used a mean excitatory and inhibitory strength of 5 and -6, respectively. The abscissa shows the fraction of the excitatory neurons that had modified connectivity. The ordinate gives the amount of connections added, defined as a fraction of the neuron’s original number of outputs. (**B**) The same type of information presented in panel (A), except here shown for a network that had a mean excitatory strength of 7 and a mean inhibitory strength of −6. (**C**) The fraction of autaptic conditions (fraction of neurons x amount of autapses; see Methods) within a given excitation and inhibition level that were significantly different from the original baseline network and from the corresponding input and output null models when autapses are added to excitatory (left) versus inhibitory (right) neurons. Bar graphs show the fraction of conditions that were significantly different from the baseline network and from control conditions across a particular excitatory or inhibition level.

We added varying amounts of autapses or non-autaptic connections to a specified fraction of randomly selected excitatory or inhibitory neurons throughout the network (Fig. 4). At lower excitatory strengths, burst frequency increased with both the percent of autaptic neurons and the number of autapses added to autaptic neurons. However, adding non-autaptic connections in the null model simulations did not increase burst frequency (Fig. 4A). At higher excitatory strengths, burst frequency again increased with the percent of autaptic neurons and the number of autapses added (Fig. 4B). Here, unlike at lower excitation levels, adding non-autaptic connections in the null model simulations also increased bursting frequency; however, higher levels of bursting still occurred in the autaptic network compared to the null model networks.

We can summarize the data described above by computing the fraction of autaptic conditions (fraction of neurons x amount of autapses; see Methods) within each excitation and inhibition combination that had significantly different burst frequencies from those observed in the original non-autaptic system and from the burst frequencies of the corresponding null model systems (Fig. 4C). We observed that adding autapses to excitatory neurons induces greater changes in burst frequency than adding autapses to inhibitory neurons. Moreover, we observed that, when adding autapses to excitatory neurons, the level of excitation more strongly affects changes in the network’s bursting behavior than the level of inhibition.

**Figure 5:**
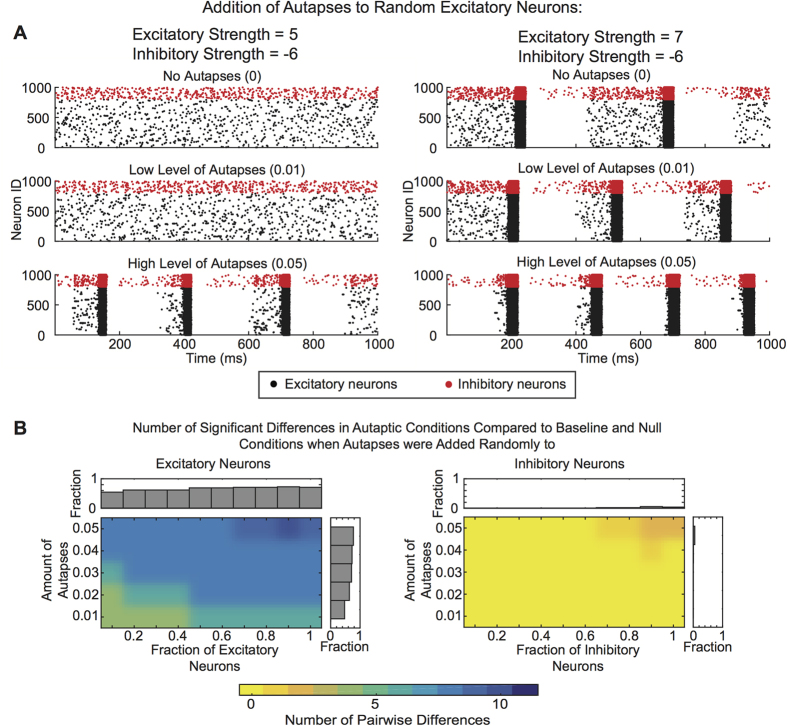
Effect of Amount of Autapses on Network Dynamics. (**A**) Raster plots showing 1 s of activity for networks with lower (left) and higher (right) excitation levels, as the amount of autpases is varied from 0% (top) to 5% (bottom). (**B**) The fraction of excitatory and inhibitory strength pairs at which the bursting frequency after addition of autapses was significantly different from the bursting frequency at baseline or in the null models. Number of pairwise differences are given as a function of the amount of autapses added, as well as the fraction of excitatory (left) and inhibitory (right) neurons. Bar graphs show the fraction of excitatory/inhibitory strength pairs that produced bursting frequencies that were significantly different from the baseline network and control conditions across the amount of autapses added.

Next we asked how these results depended on the number of autaptic connections that were added to the network. Networks constructed with more autapses displayed an increased burst frequency and burst regularity compared to networks constructed with fewer autapses (Fig. 5A). Interestingly, the relationship between burst frequency and number of autapses was modulated by the mean excitation strength of the system. At lower excitation levels, more autapses were needed to induce the same differences in burst frequency observed at higher excitation levels.

Importantly, these results do not address the question of whether the important driver of bursting dynamics is simply the number of autaptic connections, or whether the more fundamental parameter is the number of autapses per neuron. To directly address this question, we computed, for each autaptic condition (fraction of neurons x amount of autapses), the fraction of the excitatory/inhibitory strength combinations with burst frequencies that were significantly different from baseline and from the input and output null models. Again, we observed larger fraction of differences in bursting dynamics due to the addition of autapses to excitatory rather than to inhibitory neurons (Fig. 5B). We also observed that the amount of autapses added to an excitatory neuron played a larger role in driving the increase in burst frequency than the fraction of excitatory neurons in the network that were autaptic (Fig. 5B, bar graphs). These results demonstrate the effect of autapses on network dynamics is nonlinear because the same number of autaptic connections added to fewer (more) neurons has a greater (lesser) impact on burst frequency.

### Targeting Autapses to Control Neurons Differentially Impacts Burst Frequency

**Figure 6:**
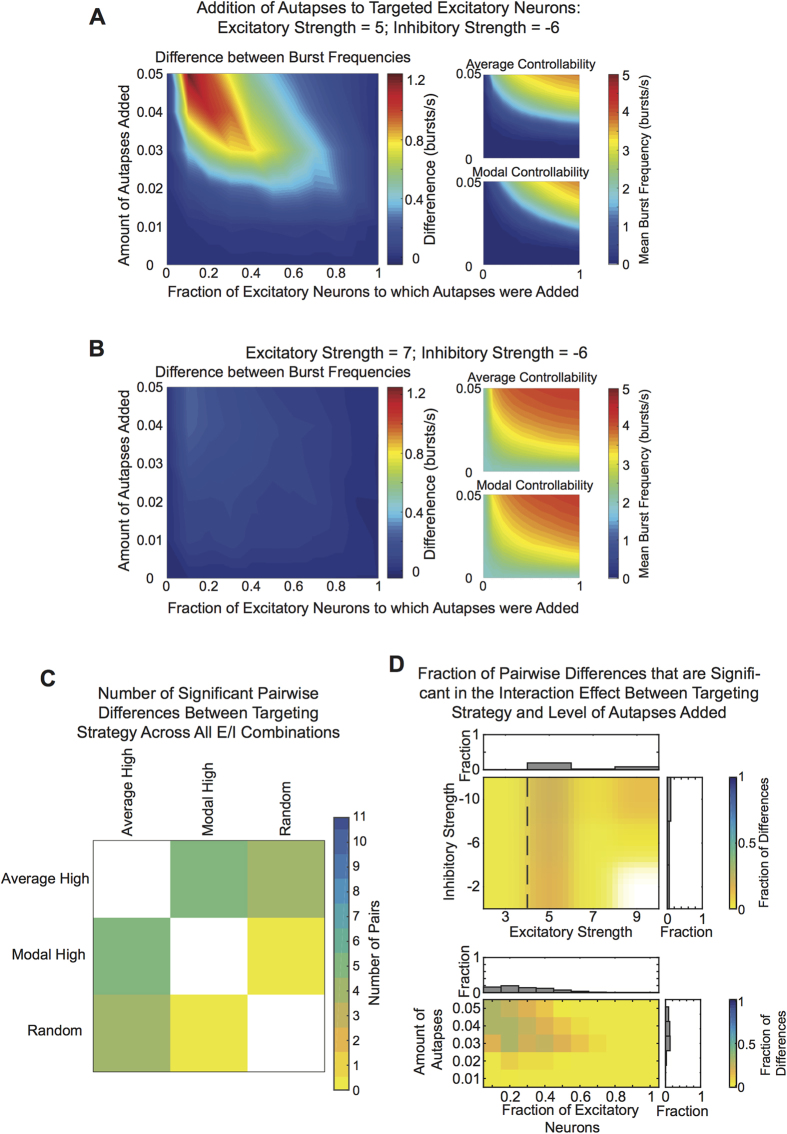
Targeting Autapses to Control Points in the Network. (**A**, left) Difference in the burst frequency when autapses were added to the highest average controllability neurons versus the highest modal controllability neurons, as a function of the fraction of excitatory neurons to which autapses were added, for example simulations. (A, right) Observed burst frequency when autapses were added to the highest average (top) versus highest modal (bottom) controllability neurons for example simulations. The abscissa shows the fraction of the excitatory neurons to which autapses were added. The ordinate gives the amount of connections added, defined as a fraction of the neuron’s original number of outputs. (**B**) Similar data to that presented in panel (A) except here for a higher level of excitation. (**C**) Number of significant pairwise differences – across all eleven excitation and inhibition combinations – in burst frequency for networks constructed from the two targeting strategies. (**D**, top) Fraction of significant pairwise differences in burst frequency in the interaction effect between targeting strategy and amount of autapses added, within each excitatory/inhibitory strength combination. (D, bottom) Fraction of pairwise differences in burst frequency across excitatory and inhibitory strength combinations at each autaptic condition (fraction of neurons x amount of autapses). Bar graphs show the fraction of excitatory/inhibitory strength pairs that produced bursting frequencies that were significantly different between the targeting strategies across the amount of autapses added.

After studying the effect of autapses added to neurons chosen uniformly at random, we next asked whether we could target autapses to specific “control” neurons to increase burst frequency even further. To address this question, we examine bursting dynamics when autapses are added to either excitatory or inhibitory neurons with either the highest average or highest modal controllability values (for definitions, see Methods). At a mean excitatory strength of 5, adding autapses to excitatory neurons with the highest average controllability resulted in higher burst frequencies at certain autaptic conditions than when autapses were added according to the highest modal controllability (Fig. 6A). At a stronger excitatory level of 7, although there was a significant interaction effect between the amount of connections added and the targeting strategy, we did not observe significant differences between corresponding autapse levels of average and modal controllability (Fig. 6B).

To better understand the impact of excitatory and inhibitory strength values on these results, we calculated the number of significant differences in burst frequency between average and modal controllability targeting strategies that occurred across the 11 excitatory and inhibitory strength combinations (Fig. 6C). We observed a maximum of 5 differences, which can be explained by Fig. 5D where we see that significant pairwise differences between bursting dynamics observed in different targeting strategies only occurred at excitation/inhibition levels of 5/−2, 5/−6, 5/−10, 7/−10 and 9/−10. Additionally, the majority of significant differences between targeting strategies occurred when autapses were added to less than half of the excitatory neurons (Fig. 6D). As we added autapses to increasingly more neurons according to these two opposing targeting strategies, the overlap between the groups of neurons that targeting strategies selected increased, making the rules more similar and leading to fewer observed differences in bursting dynamics.

Results from all targeting strategies are shown in Supplementary Information. No targeting strategy or interaction effect was observed when we added autapses to inhibitory neurons.

## Discussion

Here we examine the relationship between theoretical measures of structural controllability and observed measures of network dynamics. We build on a well-developed numerical simulation of cortical and hippocampal network dynamics to study the influence of autaptic connections on bursting frequency and regularity. Autaptic connections are represented as self-loops in the network and present unique control features whose impact on neuronal network dynamics is unknown. We show that these self-loops differentially influence network dynamics: when applied to excitatory (but not inhibitory) neurons, these self-loops lower the threshold for network bursting. Directing self-loops to nodes of high average controllability, which are theoretically predicted to effectively move the system into local easily-reachable states, leads to an increase in the frequency and regularity of network-wide bursts. Together, these results suggest a role of autaptic connections in controlling network-wide bursts in diverse cortical and subcortical regions of mammalian brain.

### Dynamic Behaviors Driven by Structural Network Architecture

In our network, the most salient outputs are the appearance of bursting or synchronization of the network, and the corresponding interburst intervals, that appear over time. Synchronization of brain networks is often considered to be key for learning^52^, memory^53^,^54^, and other higher-order cognitive processes^55^,^56^,^57^. In contrast, sporadic or sustained bursting can lead to the development of pathological networks in diseases such as epilepsy^58^. Our findings show that bursting will appear over some, but not all, combinations of excitatory/inhibitory synaptic strength combinations. Our results describing a broad class of bursting types are consistent with previous models showing a dynamic range of activity in neuronal systems, including the coherent activity observed in health and the abnormal activity observed in disease. These results further add to the literature by demonstrating that the observed dynamics (burst frequency and regularity) are directly driven by the underlying network connectivity and synaptic weights between neurons. These networks were designed to model only local microcircuit architectures with no delay among neurons in the network. Our findings provide insight into how these local self-loops can regulate the neural dynamics of these microcircuits. A critical feature for the influence of self-loops is that the circuits exist near or above the transition for bursting behavior. At low synaptic strength levels, adding autaptic connections did not elicit a bursting phenomenon because the network simply required more synaptic input than the autaptic connections provided to fire. At or near the transition for bursting, we found that spreading autaptic connections among a number of excitatory neurons affected the output neural dynamics (bursting) more significantly than concentrating many autapses to a smaller number of neurons. From a network perspective, this general result indicates that drivers of network behavior exist preferentially at the level of single nodes, rather than at the level of single edges within the network. The observation of single node drivers persisted across networks of different sizes, whereas adding edges would affect the relative transition to bursting behavior.

### **A**utapses as Effective Drivers of Shifting Network Dynamics

Physiological estimates of autaptic connections in excitatory neurons rarely exceed 1–2% of neurons within a network^59^, while some interneurons can display significant levels of self-inhibition^22^,^23^,^24^. It is interesting to note that we observed significant transitions network-wide behavior when our simulations extended beyond these physiological conditions. These data support the plausible intuition that neuronal networks *in vivo* operate at an optimal point for shifting network dynamics by the deletion or the addition of only a few self-loops, supporting maximal flexibility or dynamic range. This type of self-loop modulation might occur as a function of synaptic pruning that is common during neuronal development, which can work to consolidate network dynamics towards a stable equilibrium point^60^. An alternative potential mechanism for self-loop formation is sprouting, commonly observed after injury, which could transform a low activity network into a highly active network with periods of synchronization^61^. The impact of these dynamics are less clear and depend on the frequency of the neuronal activity, with an enhancement of activity potentially promoting prosurvival signaling through the nuclear activation of antioxidant signaling pathways^62^,^63^. Alternatively, extensive aberrant sprouting could drive the network into a state of overexcitation, which could in turn lead to targeted neurodegeneration from chronic, seizure-like bursting of the network.

### **L**inear Predictions of Nonlinear Dynamics

A key question we explored was how the nonlinear dynamics of this commonly studied network were influenced by the patterns of structural connections surrounding single neurons. To gain an understanding of this relationship, we drew from the field of structural controllability^64^,^65^,^66^: a subfield of control and dynamical systems theory that offers predictions of which nodes in a network might act as control points under the assumptions of a simplified linear dynamics. We asked whether these predictions offered fundamental utility in understanding the complex behaviors of neuronal networks. We observed that for a range of excitatory and inhibitory synaptic strengths, the structural network change elicited by adding autapses to putative control points in the network increased burst frequency and regularity, to a much greater degree than adding autapses to neurons chosen uniformly at random. These results demonstrate that the predictions of control points derived from a simplified linear model of neuronal network dynamics are supported by observed changes in network dynamics, consistent with reported results at larger spatial scales^49^. It will be interesting in future to study the role of alternative control strategies (including boundary controllability^19^,^42^) in the other areas of the excitation/inhibition phase space characterized by other network behaviors including either continuous bursting or the lack of bursting.

### Inference of network dynamics through controllability

Although we observed correspondence between measures of controllability and the resulting dynamical behavior of networks, we recognize the activation and bursting phenomenon that appears in the networks is a nonlinear process. As the linear approximation of inherent nonlinear systems is under active study, we found many corresponding connections between linear control theory and bursting behavior, but these were not complete. For example, moving a network that is already bursting into a higher bursting state may be viewed as an easily approachable new state, and control theory would predict nodes with high average controllability would be ideal for moving the network into this new state. This is consistent with our observations, as excitatory neurons represented neurons of high average controllability in these networks and adding autaptic connections specifically to these neurons moved the network into a higher bursting state. Likewise, a network that is currently not bursting can reach another easily reachable non-bursting state by adding autaptic connections to inhibitory neurons, as these neurons represent a majority of the neurons with high average controllability. In comparison, shifting a network into a difficult to reach state – e.g., moving a bursting network into a non-bursting network – suggests that neurons with high modal controllability would be the likely targets. However, in such a network, the inhibitory neurons represented nearly all of the nodes with high modal controllability and adding autaptic connections did little to affect the dynamics. Although this may highlight one gap in using linear control theory to predict nonlinear dynamics of neural networks, it is worth noting that we could shift bursting networks into nonbursting networks at higher levels of inhibitory synaptic strength, suggesting at least a regime of the network where the predictions align across the two domains.

### Methodological Considerations

There are several important limitations to this work that could be explored in future studies. First, these simulations do not provide more detailed mechanisms of network remodeling (e.g., spike timing dependent plasticity, homeostatic plasticity, presynaptic facilitation) that may affect the temporal evolution of bursting that can occur by including more detailed models of synaptic currents. However, we do not anticipate that these more detailed features of the model would affect our general result of changes in bursting dynamics associated with changes in underlying network architectures. Second, we also examined a range of excitatory and inhibitory synaptic strengths used in past studies, and found that the principal change in dynamical behavior was mediated through the excitatory neurons. Extending the current work into regimes where the inhibitory synaptic strength also influences the bursting behavior of the network would test the robustness of our observations for network controllability where alterations in either excitatory or inhibitory strength would lead to changes in the neural dynamics. Third, we directly addressed the question of whether predictions of control points in the network derived from a simplified linear model of neuronal dynamics could be used to understand the nonlinear dynamics of the full model. It would be interesting in future to develop and apply techniques from nonlinear control theory to further understand the mapping between control points and observed network dynamics. Indeed, studying nonlinear control strategies could offer particular utility in extending these examinations to the study of the switch timing of transcriptional activators and repressors within genetic circuits that code for neuronal function. Finally, the notions of average and modal controllability are agnostic to the specific initial and final states of the system, offering predictions based on the ensemble of local easily-reachable states (average controllability), and the ensemble of distance difficult-to-reach states (modal controllability). It could be interesting in future work to study specific transitions of the neuronal network from a specified initial state of activation to a specified final state of activation, potentially offering insights into the finite set of transitions that a network is expected to display under normal operating conditions^48^,^67^.

## Conclusion

In this study, we focus predominantly on descriptive statistics and simple predictors of future network performance and dynamic behavior. However, the time is ripe for the field of network neuroscience to take the next step in the *de novo* design of networks theoretically optimized for specific types of computations. These design efforts capitalizing on generative modeling frameworks would be especially important for understanding the different structure-function mappings observed across different regions of cortex, as well as in health *versus* disease, and to posit therapeutic interventions for network reconfiguration and recovery. We anticipate that the targeted placement of autaptic connections will be an important dimension of these solutions, as well as more generally being critical for our understanding of the dynamics observed in translation, transcription, and gene regulatory networks.

## Additional Information

**How to cite this article:** Wiles, L. *et al*. Autaptic Connections Shift Network Excitability and Bursting. *Sci. Rep.* **7**, 44006; doi: 10.1038/srep44006 (2017).

**Publisher's note:** Springer Nature remains neutral with regard to jurisdictional claims in published maps and institutional affiliations.

## Supplementary Material

Supplemental Text
