# Supplementary material for: Autaptic Connections Shift Network Excitability and Bursting
Source: Sci Rep. 2017 Mar 7;7:44006. doi: 10.1038/srep44006 (PMC5339801; doi:10.1038/srep44006)
Supplement: Supplemental Text [file srep44006-s1.pdf]

Supplementary Materials

Autaptic Connections Shift Network Excitability and Bursting

Laura Wiles[1], Shi Gu[1,2], Fabio Pasqualetti[3], Brandon Parvesse[1], David Gabrieli[1], Danielle S. Bassett[1,4], David F. Meaney[1,5]

[1] Department of Bioengineering, School of Engineering and Applied Sciences, University of Pennsylvania, Philadelphia, PA 19104 USA

[2] Applied Mathematics and Computational Science Graduate Program, School of Arts and Sciences, University of Pennsylvania, Philadelphia, PA 19104 USA

[3] Department of Mechanical Engineering, University of California Riverside, CA USA

[4] Department of Electrical and Systems Engineering, School of Engineering and Applied Sciences, University of Pennsylvania, Philadelphia, PA 19104 USA

[5] Department of Neurosurgery, Perelman School of Medicine, University of Pennsylvania, Philadelphia, PA 19104 USA

Section 1: Supplementary Results

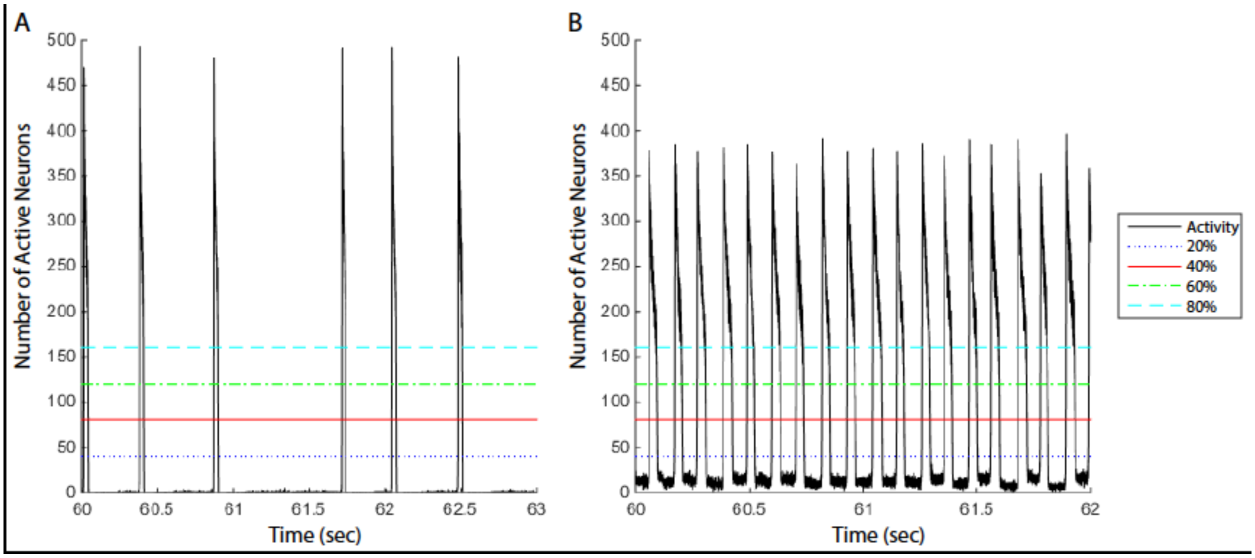

Supplementary Figure 1: Robustness of results to burst detection threshold. In the main manuscript, we define network-wide bursts as periods of activity in which the number of neurons firing at the same time met or exceeded a threshold level of 40% of neurons in the network divided by the number of steps per ms. Here we show that our results were robust to changes in the threshold level of neurons that must be active for a period of activity to be considered a burst. (A) Number of active neurons as a function of time in seconds for an example simulation at the excitatory strength of 7. (B) Number of active neurons as a function of time in seconds for an example simulation at the excitatory strength of 9. Colored horizontal lines indicate different threshold choices (20%, dark blue dotted line; 40%, red solid line; 60% green dot-dashed line; 80%, cyan dashed line). Note that the number of network-wide bursts is identical across these threshold choices.

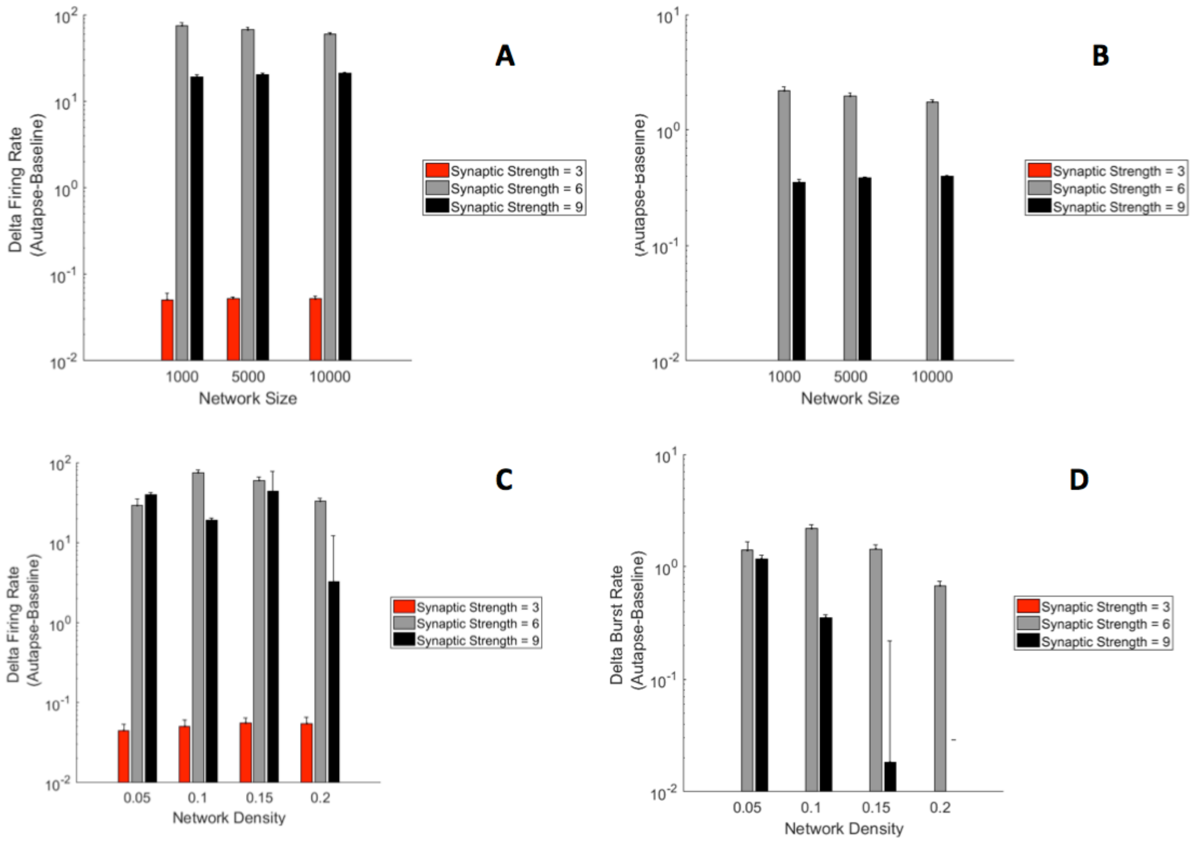

Supplementary Figure 2: Network size does not affect activity dynamics, but network density can lead to an alteration in firing rate. A. Across networks sizes that could be as large as 10,000 total neurons, the relative change in firing rate across different excitatory synaptic strengths did not differ significantly. B. Similarly, when autapses were added to the same fraction of neurons across these different-sized networks, there was no significant change in the burst rate differences that appear across these networks. C. In comparison, adding more connections of similar synaptic strength to increase the network density would lead to more active networks. However, the difference in firing rates that occurred when autapses were added in these increasingly dense networks would not change significantly until the networks started to show bursting behavior in the initial state (density .15, .20). D. For these more dense networks, bursting became more common at high synaptic strengths (9) and, in turn, led to no significant difference in the change of bursting rate when autapses were added.

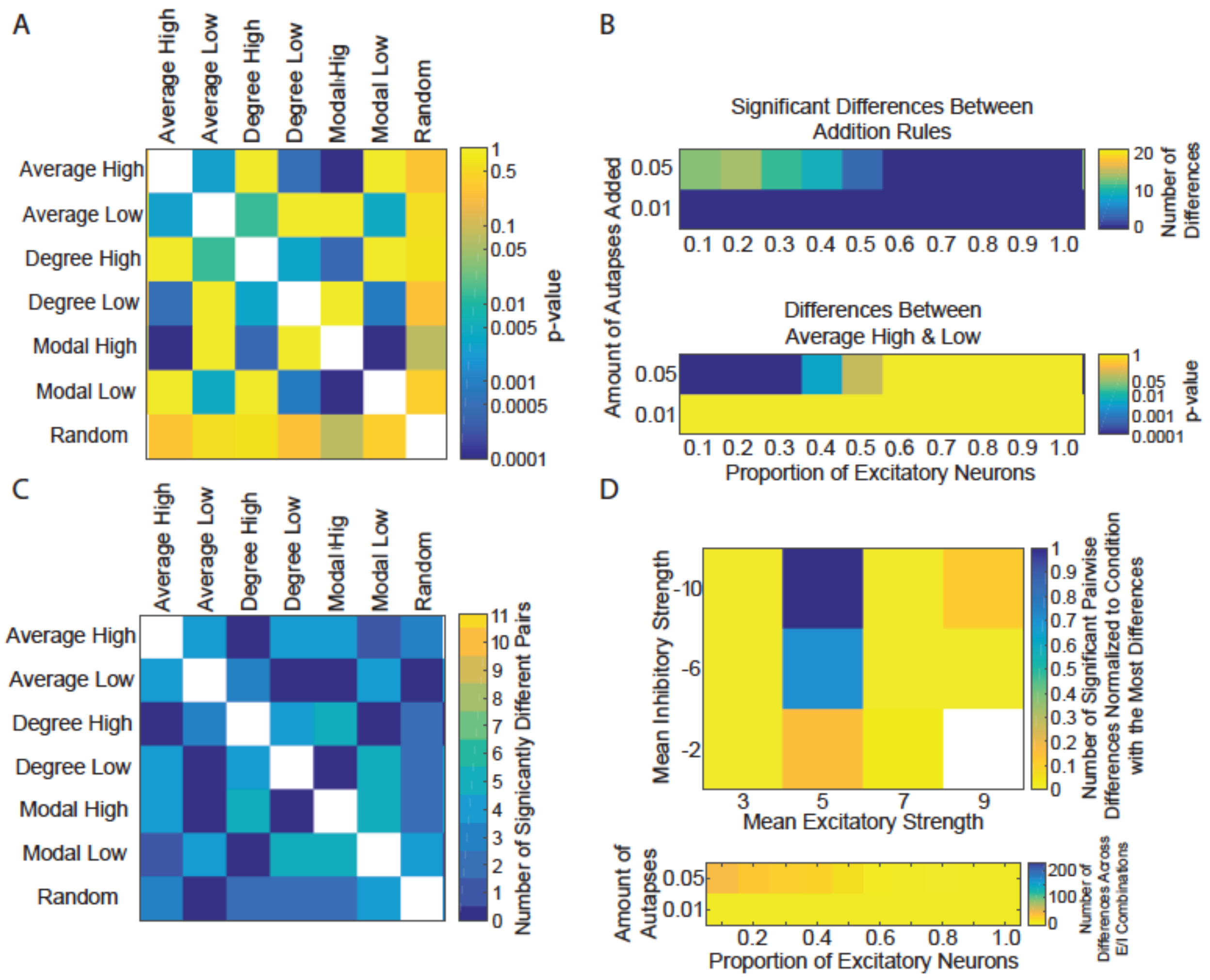

Supplementary Figure 3: Effect of targeting strategy. In the main text, we report results comparing three targeting strategies using a mixed-model ANOVA: neurons chosen by highest average controllability, highest modal controllability, and uniformly at random. Here, we use the same statistical procedure to show results for all seven targeting strategies. (A) Differences in burst frequency induced by adding connections according to the seven targeting strategies, Color indicates p-values of posthoc Tukey's HSD test. (B) Dependence of results in (A) as a function of both the proportion of excitatory neurons (x-axis) and the amount of autapses added (y-axis). The number of significant differences between addition rules was greater for simulations with more autapses and smaller proportion of excitatory neurons. (C) Number of significantly different pairs of simulations (across different levels of excitatory and inhibitory strength, and across different amounts of autapses) for each set of targeting strategies. Color

indicates number of significantly different pairs. (D) Dependence of summary results in panel (C) on the inhibitory/excitatory strength combination (top) and the amount of autapses added (bottom).

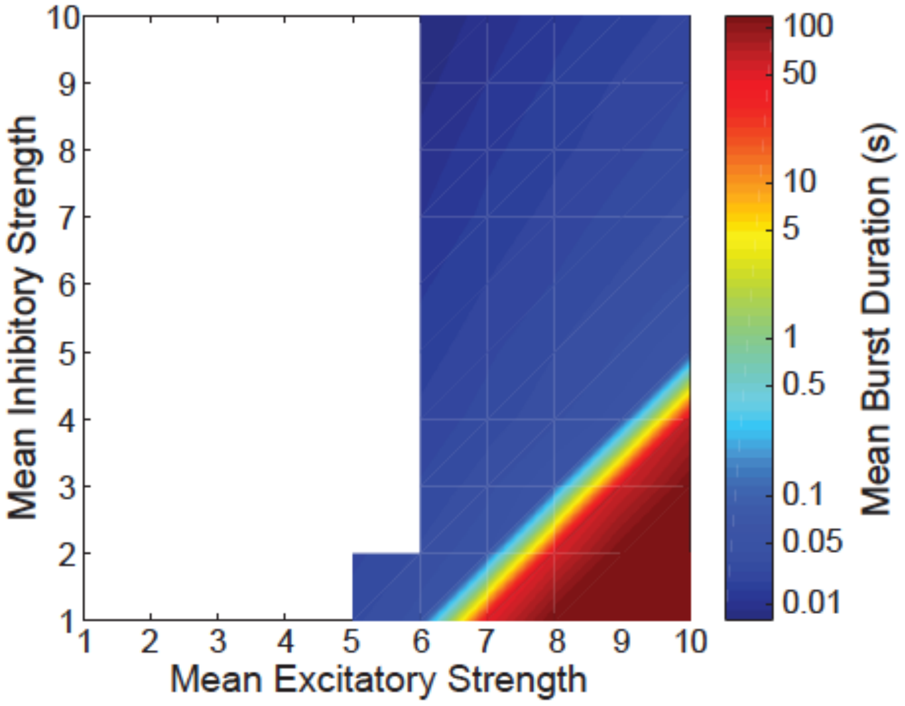

Supplementary Figure 4: Burst duration dependent on excitatory and inhibitory strength. Mean burst duration (color) as a function of mean excitatory strength (x-axis) and mean inhibitory strength (y-axis).

Section 2: Supplementary Discussion

In the main manuscript, we utilize a mathematical definition of a network-wide bursts. However, it is important to note that there is no one well-accepted definition of a network-wide burst. To illustrate this point, here we describe previously-used definitions, which vary both quantitatively and qualitatively.

1. In a previous computational model, the resulting spiking activity was binned into 10 ms time windows. A burst was identified when more than 25% of the neuronal population fired during that time window [1].

2. In a different study that included both a computational model and multi-unit activity (MUA) from multielectrode array recordings of *in vitro* rat cortical neurons, a "network spike" was detected by counting the spikes recorded at all electrodes in 50 ms time windows. If the number of spikes detected exceeded a threshold of 25% of the maximum spike count recorded, then a burst was detected [2].

3. In another analysis of MUA activity, recordings from each electrode were searched for sequences of sequences of at least four spikes with inter-spike intervals less than a threshold set to the less of 100 ms or ¼ of that electrode's inverse spike detection rate. If a group of these sequences overlapped in time across multiple electrodes, it was called a burst [3].

4. Pasquale et al [4] developed a burst detection algorithm involving two cases based on the separation of the two peaks (bursting ISI peak and non-bursting ISI peak) logarithmic histogram of the ISI. If the minimum between the two peaks is less than 100 ms, that minimum is used as the maximum ISI allowed within a burst and used to identify spikes within a burst. On the other hand, if the minimum is greater than 100 ms, a 100 ms threshold is used to detect burst cores and the location of the minimum between the peaks is used as the absolute bursting threshold. Spikes occurring within the burst core ISI threshold are identified as burst core spikes while spikes occurring at rates between the burst core ISI threshold and the minimum on the histogram are referred to as burst boundary spikes.

5. Recent work utilized and compared the results of several burst detection algorithms. These included: (a) a rate-threshold detector in which a burst was detected if a firing rate histogram with 50 ms time windows exceeded a threshold number of spikes, (b) a ISI-threshold detector in which interspike interval thresholds were set at the minimum between peaks of the logarithmic ISI probability distribution and capped at 10 ms. If the ISIs of five consecutive spikes were each less than the ISI threshold, then a single-channel burst was detected. A network burst required 20% of the recording channels to be activated. (c) a rank surprise detector in which the minimum number of spikes and maximum ISI within a burst were 5 spikes and 100 ms, respectively, and at least 10 single channel bursts overlapped in time, and (d) an ISI_N-threshold method in which the user chooses a minimum network burst size in terms of N, the number of spikes that

make the smallest network burst, and then if N consecutive spikes occur within a time period less than or equal to the ISI_N-threshold, where ISI_N is the ISI between every Nth spike in the network and the ISI_N-threshold is the minimum between regions of bursting and non-bursting in a logarithmic histogram of ISI_N, the time period is a burst [5].

References:

1. Luccioli, S., et al., *Clique of functional hubs orchestrates population bursts in developmentally regulated neural networks.* PLoS Comput Biol, 2014. **10**(9): p. e1003823.
2. Masquelier, T. and G. Deco, *Network bursting dynamics in excitatory cortical neuron cultures results from the combination of different adaptive mechanism.* PloS one, 2013. **8**(10): p. e75824.
3. Wagenaar, D.A., Z. Nadasdy, and S.M. Potter, *Persistent dynamic attractors in activity patterns of cultured neuronal networks.* Physical Review E, 2006. **73**(5): p. 051907.
4. Pasquale, V., S. Martinoia, and M. Chiappalone, *A self-adapting approach for the detection of bursts and network bursts in neuronal cultures.* Journal of computational neuroscience, 2010. **29**(1-2): p. 213-229.
5. Bakkum, D.J., et al., *Parameters for burst detection.* Frontiers in computational neuroscience, 2014. **7**: p. 193.
